# The Dynamics of Interleukin-10-Afforded Protection during Dextran Sulfate Sodium-Induced Colitis

**DOI:** 10.3389/fimmu.2018.00400

**Published:** 2018-03-01

**Authors:** Ana Cardoso, Antonio Gil Castro, Ana Catarina Martins, Guilhermina M. Carriche, Valentine Murigneux, Isabel Castro, Ana Cumano, Paulo Vieira, Margarida Saraiva

**Affiliations:** ^1^i3S – Instituto de Investigação e Inovação em Saúde, Porto, Portugal; ^2^IBMC – Instituto de Biologia Molecular e Celular, Universidade do Porto, Porto, Portugal; ^3^Department of Immunology, Unité Lymphopoièse, Institut Pasteur, Paris, France; ^4^University Paris Diderot, Sorbonne Paris Cité, Cellule Pasteur, Paris, France; ^5^INSERM U1223, Paris, France; ^6^ICVS, University of Minho, Braga, Portugal; ^7^ICVS/3B’s – PT Government Associate Laboratory, Braga, Portugal; ^8^Department of Immunology, Unité Intégrité du génome, immunité et cancer, Institut Pasteur, Paris, France; ^9^Department of Genomes and Genetics, Unité Intégrité du génome, immunité et cancer, Institut Pasteur, Paris, France

**Keywords:** interleukin-10, macrophages, inflammation, colitis, therapy

## Abstract

Inflammatory bowel disease encompasses a group of chronic-inflammatory conditions of the colon and small intestine. These conditions are characterized by exacerbated inflammation of the organ that greatly affects the quality of life of patients. Molecular mechanisms counteracting this hyperinflammatory status of the gut offer strategies for therapeutic intervention. Among these regulatory molecules is the anti-inflammatory cytokine interleukin (IL)-10, as shown in mice and humans. Indeed, IL-10 signaling, particularly in macrophages, is essential for intestinal homeostasis. We sought to investigate the temporal profile of IL-10-mediated protection during chemical colitis and which were the underlying mechanisms. Using a novel mouse model of inducible IL-10 overexpression (pMT-10), described here, we show that mice preconditioned with IL-10 for 8 days before dextran sulfate sodium (DSS) administration developed a milder colitic phenotype. In IL-10-induced colitic mice, Ly6C cells isolated from the *lamina propria* showed a decreased inflammatory profile. Because our mouse model leads to transcription of the IL-10 transgene in the bone marrow and elevated seric IL-10 concentration, we investigated whether IL-10 could imprint immune cells in a long-lasting way, thus conferring sustained protection to colitis. We show that this was not the case, as IL-10-afforded protection was only observed if IL-10 induction immediately preceded DSS-mediated colitis. Thus, despite the protection afforded by IL-10 in colitis, novel strategies are required, specifically to achieve long-lasting protection.

## Introduction

Inflammatory bowel disease (IBD) comprises a complex group of inflammatory conditions of the gastrointestinal tract (1) affecting an increasing number of patients worldwide (2–4). Both forms of IBD, Crohn’s disease (CD) and ulcerative colitis (UC), result from alterations in the immune homeostasis of the intestinal tissue leading to local uncontrolled inflammation (5, 6). The gut is a very particular site in terms of immune repertoire and regulation, as even in homeostatic conditions constant exposure to antigens occurs (7). Thus, the maintenance of intestinal homeostasis, primarily carried out by intestinal macrophages, requires a constant and fine-tuned balance between the state of tolerance and inflammation (8). In the gut environment, macrophages encounter a plethora of stimuli, from dietary antigens to commensal bacteria, yet, due to their unique tissue-specific characteristics, remain tolerant (9). In the predisease stage, the epithelial or mucosal barriers become compromised allowing bacteria from the luminal side to invade the *lamina propria* of the gut (10). This event triggers an acute inflammatory response due to the activation of immune cells by direct contact with bacterial products (10). The induced inflammation results either in elimination of the foreign bacterial incursion or in an exacerbated immune response that can result in tissue damage. The damage caused by deregulated inflammation will perpetuate the activation of effector cells and ultimately lead to the clinical onset of IBD (10, 11).

Epidemiological studies have shown that the etiology of IBD is multifactorial, with genetic predisposition, dysfunctional intestinal barrier and imbalances of the microbiome all contributing to this condition (12–15). Genome-wide association studies revealed that the main genetic alterations associated with IBD are found in genes encoding proteins linked to innate or adaptive immunity, such as the nucleotide-binding oligomerization domain-containing protein 2, Janus kinase (JAK) 2, and tumor necrosis factor superfamily 15 (16–18). Other alterations are associated with molecules involved in leukocyte trafficking, regulation of barrier function and secretion of defensins (17). Two reports associate loss-of-function mutations in interleukin (IL)-10 or IL-10R subunits with severe IBD (19, 20). These mutations result in severe enterocolitis, with onset before one year of age, and unresponsiveness to immunosuppressive therapies. The only available therapy for these patients is immune reconstitution with hematopoietic stem cells (21–23). Although complete loss-of-function mutations in IL-10 and IL-10R strongly correlate with IBD, they have an extremely low occurrence rate (19, 24). The most frequent mutations affecting the IL-10 genes associated with IBD are in fact single-nucleotide polymorphisms associated with low expression of this molecule (25). However, harboring such mutations does not always translate in low serum levels of IL-10 (23) during the disease stage. This is likely due to the significant increase on the number of IL-10-producing myeloid cells in CD patients (26–29), to the extent that elevated serum levels of IL-10 correlate with disease activity in CD (30–32).

The role of IL-10 in intestinal inflammation is also seen in the mouse model, as IL-10-deficient mice develop microbiome-dependent spontaneous enterocolitis (33). Furthermore, mice with macrophage restricted IL-10R deficiency also develop a spontaneous colitic profile (34), stressing the critical role of the monocyte/macrophage axis in the immunologic events leading to IBD. Interestingly, it has been shown, in a model of infection that IL-10 can exert a direct effect on monocytes/macrophages subsets, leading to changes in their inflammatory profile and survival (35). Moreover, IL-10 has been shown to confer protection from hyperinflammatory states by the induction of the JAK1/STAT3 signaling pathway that suppresses expression of proinflammatory mediators and activates expression of anti-inflammatory genes (36).

Taking into account the results obtained in murine models of IL-10 perturbation, the genetic correlation established in humans, and the anti-inflammatory properties of IL-10, this cytokine emerged as a very promising candidate for IBD therapy. However, in IBD patients IL-10-based therapy has not resulted in substantial clinical improvements (37). The main caveats in these clinical trials were the subcutaneous route of administration and the concentration of the recombinant molecule that did not ensure that IL-10 levels reached the mucosal sites, pointing out the importance of novel-locally targeted therapeutic strategies. Furthermore, IL-10 administration to IL-10-deficient murine models only protected from colitis if administered before disease establishment (38).

In this study, we report a novel mouse model of IL-10 overexpression (the pMT-10 mouse) and use it to better explore the mechanisms of immune regulation elicited by IL-10 in the context of intestinal inflammation. We show that a short period of IL-10 overexpression prior to the induction of colitis ameliorates the disease outcome, despite the presence of CD11b^+^ Ly6C^+^ cells in the gut, previously associated with the development of detrimental inflammation. As compared to control animals that do not overexpress IL-10, Ly6C cells isolated from the gut *lamina propria* of colitic pMT-10 mice showed a decreased inflammatory profile. Thus, we propose that IL-10 overexpression impaired the response of these cells to the stimulus. In addition to the local effect of IL-10 in controlling exacerbated immune responses, our model allows for the study of IL-10 in imprinting *de novo* generated and circulating monocytes. This is because, constant IL-10 expression is found in specific tissues, in pMT-10 mice, culminating in a systemic effect. Therefore, IL-10 is likely to affect other important compartments, such as the bone marrow (BM) and spleen. IL-10-afforded protection was only seen if IL-10 triggering immediately preceded dextran sulfate sodium (DSS)-induced colitis, thus calling for novel strategies that sustain the effect of IL-10 to offer long-lasting protection.

## Materials and Methods

### Ethics Statement

In Portugal, all animal experiments were performed in strict accordance with recommendations of the European Union Directive 2010/63/EU and previously approved by Portuguese National Authority for Animal Health–Direção Geral de Alimentação e Veterinária (DGAV). Mice were euthanized by CO_2_ inhalation with efforts to minimize suffering.

In France, all animal procedures were approved by the Pasteur Institute Safety Committee and conducted according to French and European Community Institutional guidelines.

### Animals

The study involved the use of the following 7–14-week-old female mice: wild-type C57BL/6j, pMT-10-IL-10 inducible mice, and pMT-10 crossed with IL-10Rα-deficient mice (39) (pMT-10.IL-10Rα^−/−^). Food was *ad libitum* for all animals.

### Generation of pMT-10 Mice

**Figure 1:**
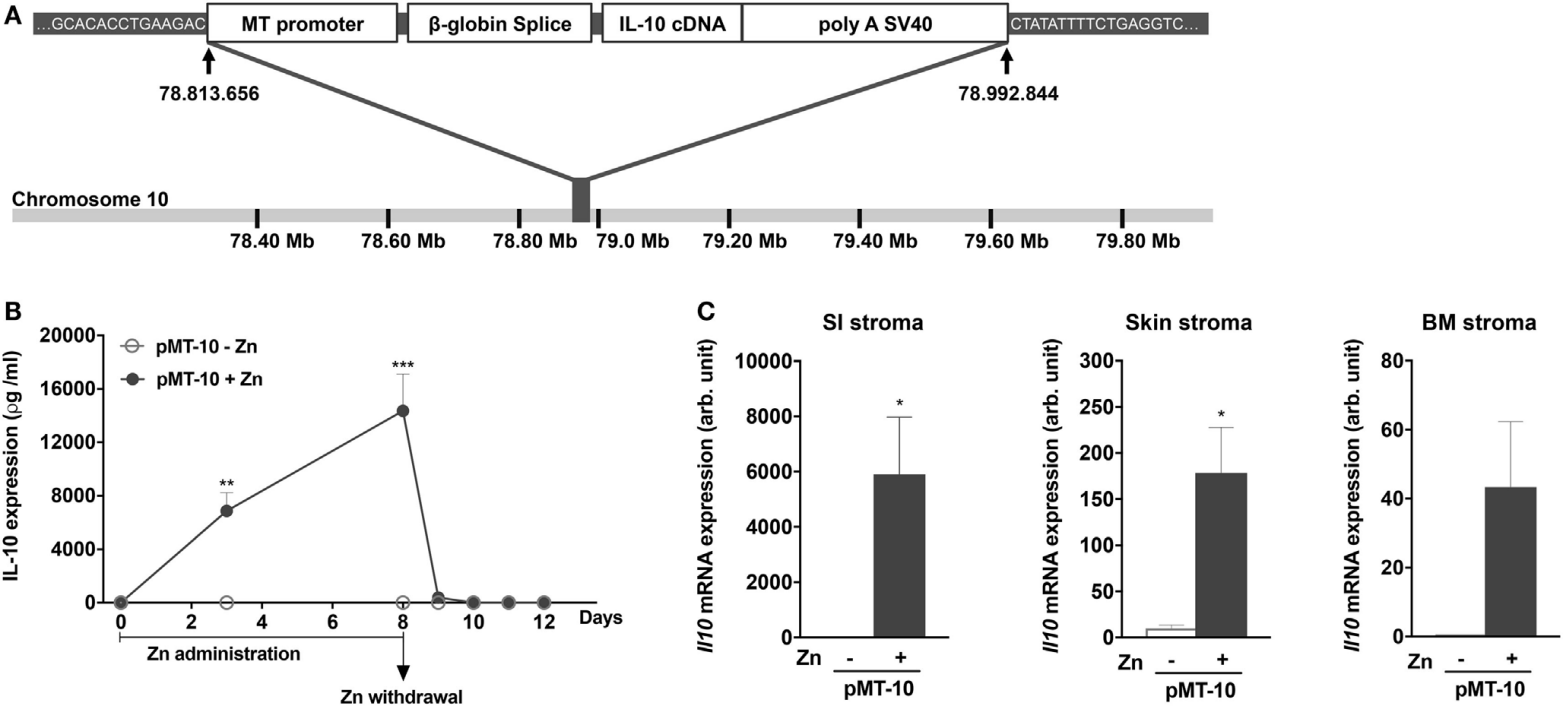
A novel mouse model for inducible interleukin (IL)-10 expression: pMT-10 mice. **(A)** Schematic representation showing the targeting vector and insertion site. **(B)** Kinetics of IL-10 overexpression in the serum at different time points post Zn administration and Zn withdrawal. pMT-10 mice were fed with normal (pMT-10-Zn) or Zn-enriched (pMT-10 + Zn) water and at the indicated time points blood was harvested and the amount of IL-10 in serum measured by immunoassay. **(C)** qRT-PCR identified CD45^−^ TER119^−^ cell subsets from skin, bone marrow, and small intestine (SI) as the main producers of IL-10 in pMT-10 mice fed for 8 days with Zn-enriched water. In both **(B,C)**, each point or bar represents the mean ± SEM for three independent mice. Data were analyzed with **(B)** two-way analysis of variance (Sidak’s multiple comparisons test) or **(C)** Student’s *t*-test, *p < 0.05; **p < 0.01; ***p < 0.001.

pMT10 mice were generated by A. Gil Castro and Paulo Vieira. Mouse IL-10 cDNA was cloned into the p169ZT vector, which carries a sheep metalloprotein (MT) 1a promoter, a β-globin splice site and a SV40 polyadenylation (polyA) signal. The resulting vector—pMT-10 (see Figure 1A)—was then injected into C57BL/6j eggs and transgenic founders were identified by PCR using MT and IL-10-specific primers. IL-10 overexpression was induced by feeding the mice a 2% sucrose solution with 50 mM of zinc (Zn) sulfate.

### DSS-Induced Colitis

Mice were fed for 8 days with 3% DSS (TdB consultancy) in the drinking water, and were monitored, daily, for weight loss and disease progression. Colitis progression was measured by the Disease Activity Index (DAI), as previously described [Table 1 (40)].

**Table 1:** Disease Activity Index (DAI) parameters.

Score	Weight loss	Stool consistency	Bleeding
0	No loss	Normal	No blood
1	1–5%	Mild soft	Brown color
2	5–10%	Very soft	Reddish color
3	10–20%	Diarrhea	Bloody stool
4	>20%		Gross bleeding

*DAI is obtained by the sum of each individual score*.

### Assessment of Intestinal Inflammation

Mice were euthanized on day 8 post-DSS administration or earlier if the symptoms of clinical disease (significant weight loss or diarrhea) became apparent. Samples from colons were immediately fixed in 4% paraformaldehyde. Then, 5 µm paraffin-embedded sections were stained with hematoxylin and eosin, and inflammation was assessed in a blinded fashion using a previously described system [Table 2 (41)]. Samples were graded semiquantitatively from 0 to 3 for the four following criteria: (i) degree of epithelial hyperplasia and goblet depletion; (ii) leukocyte infiltration in the *lamina propria*; (iii) area of tissue affected; and (iv) the presence of markers of severe inflammation such as crypt abscesses submucosal inflammation and ulcers. For each sample, criteria scores were added to give an overall inflammation score of 0–12.

**Table 2:** Parameters for histological analysis of colitis severity.

Score	Epithelial hyperplasia and goblet depletion	Leukocyte infiltration in the *Lamina Propria*	Area affected	Markers of severe inflammation
0	None	None/rare	None	None
1	Minimal	Increase	1/3	Minimal
2	Mild	Confluent	2/3	Increased
3	Marked	Transmural	All	Confluent

*The final score is obtained by the sum of individual scores. Markers of severe inflammation included ulceration and crypt abscesses*.

### Cytokine Quantification

Interleukin-10 concentration in the serum was quantified using a commercially available ELISA kit (R&D systems).

### Preparation of Cell Suspensions

Isolation of non-hematopoietic cells (CD45^−^ TER119^−^) or hematopoietic (CD45^+^) BM cells followed standard protocols. Briefly, hematopoietic BM cells were extracted by flushing the femurs and tibias with 2 mL of HBBS repeatedly. To obtain the BM non-hematopoietic cells, the bone fragments were incubated in RPMI medium with Liberase TL (0.5 mg/ml; Roche) for 30 min at 37°C. To help dissociation of non-hematopoietic cells from the bone, after each incubation period, the femurs and tibias were flushed with RPMI. We repeated this step three times after which we flushed the bones one last time, harvested the cell suspensions and added 1 volume of RPMI containing 10% FCS. Small intestine (SI), non-hematopoietic cells were isolated as previously described (42). Skin non-hematopoietic cells were isolated from ear samples. Samples were harvested and the epidermis exposed by separating the external layers. Epidermis was incubated for 45 min at 37°C with Liberase HL (0.5 mg/ml; Roche) and DNase I (1 U/mL; Invitrogen). After 45 min, non-hematopoietic cells were dissociated from the tissue by mechanical disruption, collected, washed in HBSS containing 10% FCS and recovered. At the end, non-hematopoietic cells were sort-purified by excluding all CD45^+^ TER119^+^ cells (Figure S1A in Supplementary Material). Cell suspensions for all other organs were obtained by mechanical disruption.

*Lamina propria* leukocytes (LPLs) were prepared as previously described (43). Briefly, LPLs were harvested, dissociated and resuspended in Hank’s Balanced Solution (HBSS) supplemented with 1% fetal calf serum (FCS; Gibco). To isolate LPLs, the colon was flushed with phosphate-buffered saline (PBS; Gibco), opened and cut into 1 cm pieces. To eliminate epithelial cells these fragments were incubated at 37°C in Ca- and Mg-free PBS containing 10% FCS and 5.0 mM EDTA under strong agitation for 30 min. For LPL isolation, the remaining fragments were incubated in RPMI medium with Liberase TL (0.5 mg/ml; Roche) for 30 min at 37°C. To complete the digestion, the suspension was repeatedly passed through a 10 ml syringe for 5 min, filtered through a 40 μm cell strainer (BD Bioscience) and collected by centrifugation. The cell pellet was resuspended in 44% Percoll (GE Healthcare), laid over 67% Percoll, and centrifuged at 600 *g* for 20 min at 20°C. Cells at the interface were collected, washed in HBSS containing 1% FCS and recovered.

### Antibodies

Antibodies were conjugated to fluorochromes (FITC, PE, PECy7, APC, APCCy7, Pacific Blue, and BV711) and were specific for the following mouse antigens: CD3 (145-2C11; Biolegend), CD11b (M1/70; Sony), CD11c (HL3; Biolegend), CD19 (6D5; Sony), CD45.2 (104; Biolegend), Ly6C (Hk1.4; eBioscience), Ly6G (RB6-8C5; BD Pharmingen), CD45 (30F11; Sony), and TER119 (Ter119; BD Pharmingen).

### Cell Sorting and Multiplex Real Time-PCR Analysis

CD45^−^ TER119^−^ or CD45^+^ cells were sort-purified based on the expression of CD45 and TER119 using an Aria sorter (BD). Dead cells were eliminated by exclusion with propidium iodide (PI). mRNA from sorted cells was extracted using RNeasy Micro kit (Qiagen) and converted into cDNA by reverse transcription with PrimeScript RT Reagent kit (Takara, Clonetech). qRT-PCR was performed using Taqman primers (see Table S1 in Supplementary Material for references) and Taqman Universal Master Mix (Applied Biosystems). qRT-PCR reactions were performed on a ABI 7300 thermocycler (Applied Biosystems).

Lamina propria leukocytes were FACS-sort purified based on the expression of CD45.2, CD11b and Ly6C, using an Aria III sorter (BD). Cells expressing CD3, CD19, CD11c, and Ly6G were excluded. Dead cells were eliminated by exclusion with PI. CD45.2^+^ CD11b^+^ Ly6C^+^ cells were sorted directly into a mix of 9 µl of CellsDirect One-Step qRT-PCR kit (Life Technologies), containing a mixture of diluted primers (0.05× final concentration, see Table S1 in Supplementary Material for references). Preamplified cDNA (18 cycles), was obtained according to the manufacturer’s instructions and was diluted 1:5 in TE buffer (pH = 8; Ambion). The sample mixture was as follows: diluted cDNA (2.9 µl), Sample Loading Reagent (0.32 µl; Fluidigm), and Taqman Universal PCR Master Mix (3.5 µl; Applied Biosystems). The assay mixture was as follow: Assay Loafing Reagent (Fluidigm) and Taqman Mix. A 48 × 48 Dynamic Array integrated fluidic circuit (IFC; Fluidigm) was primed with control line fluid, and the chip was loaded with assays and samples with and X IFC Controller (Fluidigm). The experiments were run on a BioMark HD (Fluidigm) for 40 cycles. Gene expression was normalized for *Hprt* and assessed by the 2Δ*^C^*^t^ method.

### Statistical Analysis

Statistical analysis was performed with the Student’s *t*-test or two-way analysis of variance as indicated in the figure legends. The analysis was performed with Prism Software (GraphPad). Graphs containing errors bars show means ± SEM. Statistical significance is represented as follows: *p < 0.05, **p < 0.01, and ***p < 0.001.

## Results

### Generation of a Novel Mouse Model of IL-10 Overexpression

To study the biological impact of IL-10 overexpression in different settings, we engineered a novel mouse model to allow for inducible IL-10 expression, the pMT-10 mouse (44). For this, a construct containing the IL-10 cDNA under the control of the inducible sheep MT promoter was introduced in the genome of wild-type BL/6 mice (Figure 1A). Whole genome sequencing revealed a single insertion of the transgene in chromosome 10, between positions 78.813.656 and 78.992844 bp (Figure 1A). We estimated, by qRT-PCR, the number of copies of the transgene to be 50–100 (data not shown). The MT promoter is activated in the presence of 50 mM of Zn in the organism, administered in the drinking water. Kinetic analysis of IL-10 in the serum of pMT-10 mice fed with Zn-enriched water showed a rapid increase of circulating IL-10 (Figure 1B). Indeed, as soon as day 3 after IL-10 induction, the levels of this cytokine in the serum were very high (7–12 ng/ml) (Figure 1B). Moreover, suspending Zn administration led to a sharp drop in IL-10 in sera in only 24 h, to below detection levels in only 48 h (Figure 1B). As expected, circulating IL-10 was undetectable in pMT-10 mice fed with normal water (Figure 1B). Transcriptional analysis of different organs and cellular compartments of induced pMT-10 mice revealed that the expression of the exogenous IL-10 cDNA was restricted to CD45^−^TER119^−^ cells from the SI, skin and, to a less extent, BM (Figure 1C). IL-10 induction was not detected in the other organs analyzed (liver, spleen, kidney, choroid plexus, lung, and colon) nor in hematopoietic cells isolated from the BM (Figure S1B in Supplementary Material). Thus, the pMT-10 mouse model allows for timely controlled IL-10 overexpression in specific anatomic locations, accompanied by a strong increase of the levels of this cytokine in the serum.

### DSS-Induced Colitis Is Ameliorated in IL-10 Preexposed Mice

Despite the clear link between low levels of IL-10 and susceptibility to colitis in human (45) as well as in mouse models (33, 34), administration of IL-10 to treat this condition showed only limited effects (37). A possible reason may be the poor accessibility of IL-10 to the site of inflammation. In this context, and in view of the high expression seen in the SI of induced mice, the pMT-10 mouse model offers an opportunity to further address the effects of IL-10 expression in the gut in the context of colitis. For this, we used the DSS experimental model, a highly reliable and reproducible way of causing UC-like symptoms in the mouse model by inducing acute inflammation with the recruitment of inflammatory cells (46).

**Figure 2:**
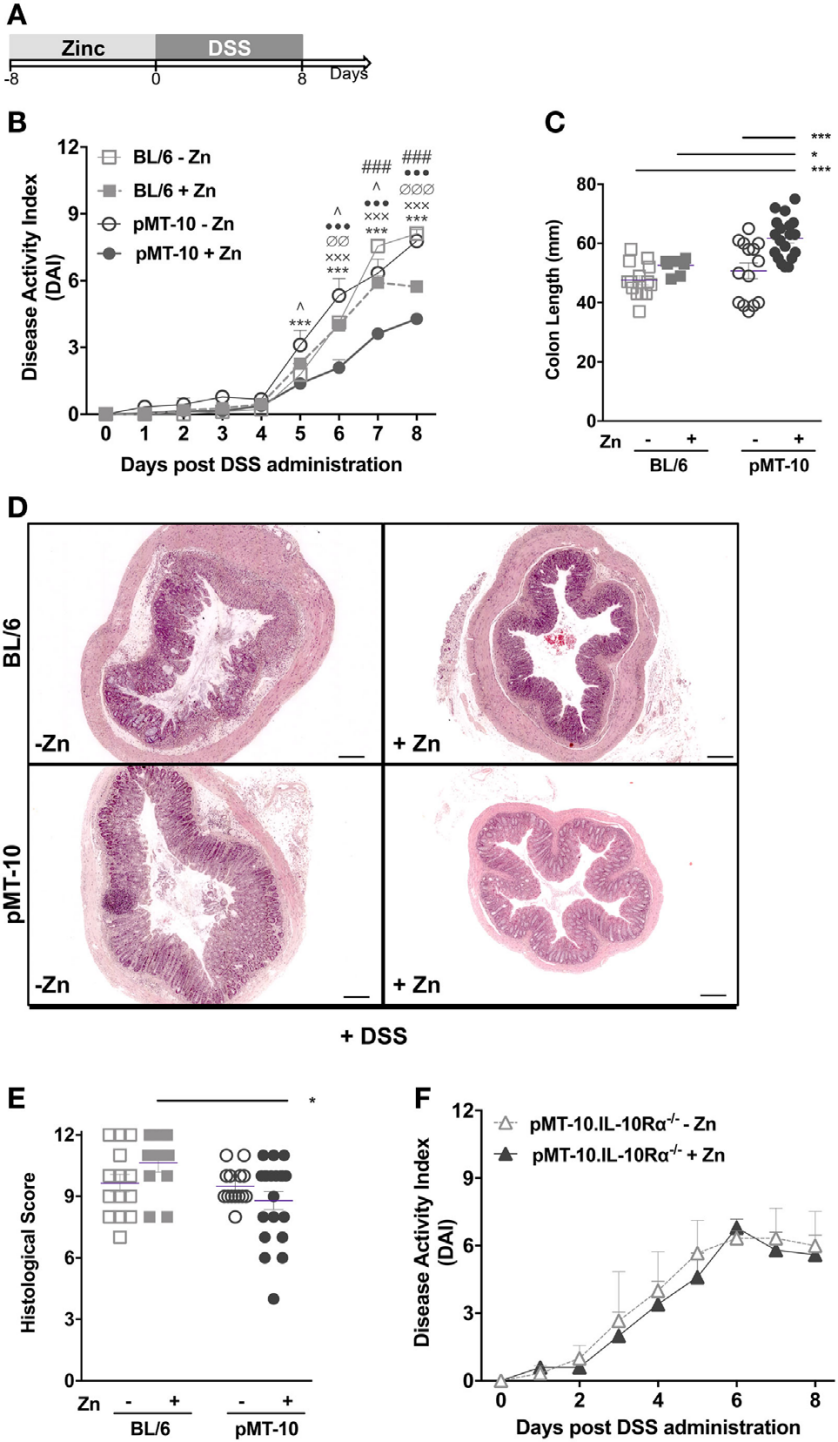
Dextran sulfate sodium (DSS)-induced pathology is ameliorated by preexposure to interleukin (IL)-10. **(A)** BL/6, pMT-10, and pMT-10.IL-10Rα^−/−^ mice were fed for 8 days with normal (BL/6-Zn, pMT-10-Zn, and pMT-10.IL-10Rα^−/−^ Zn, respectively) or Zn-enriched (BL/6 + Zn, pMT-10 + Zn, and pMT-10.IL-10Rα^−/−^ + Zn) water, followed by 8 days of 3% DSS administration also in the drinking water. **(B,F)** Disease progression based on DAI parameters was registered every day for 8 days. Each point represents the mean ± SEM for three to five independent mice, in two independent experiments. **(C)** Colon length measurement at day 8 of DSS administration. **(D)** Representative H&E-stained sections of large bowel at 40× magnification (scale bar = 200 µm). **(E)** Colitis scores derived from evaluation of colon and cecum from either group. Each dot represents one independent animal; represented is also mean ± SEM. Data were analyzed with **(B,F)** two-way analysis of variance (Sidak’s multiple comparisons test) or **(C,E)** Student’s *t*-test. **(B)** # compare BL/6-Zn against BL/6 + Zn; ^ compare BL/6-Zn against pMT-10-Zn; • compare BL/6-Zn against pMT-10 + Zn; ∅ compare BL/6 + Zn against pMT-10-Zn; x compare BL/6 + Zn against pMT-10 + Zn, * compare pMT-10-Zn against pMT-10 + Zn. One symbol, *p* < 0.05; two symbols, *p* < 0.01; three symbols, *p* < 0.001.

We investigated the impact of IL-10 overexpression prior to DSS-induced colitis. For this, pMT-10 mice were induced to overexpress IL-10 for 8 days, before initiation of DSS administration (Figure 2A). As controls, non-induced pMT-10 or BL6 mice fed with control or Zn-enriched water were used. In our experimental setting, wild-type BL/6 mice started to show signs of disease from days 4 to 5 after administration of 3% DSS in the drinking water (Figure 2B). Control pMT-10 mice showed a progression of the DAI very similar to BL/6 mice (Figure 2B). As compared to pMT-10 or BL6 fed with control water, mice preconditioned with IL-10 showed significantly lower DAI after day 5 (Figure 2B) indicating that IL-10 conferred partial protection. Zn administration to BL6 mice prior to DSS, resulted in partial protection. Indeed, by day 7 of DSS administration, a significantly lower DAI was observed in BL/6 mice fed with Zn-enriched water as compared to BL6 control (Figure 2B). Nevertheless, the maximal protection was observed for pMT-10 mice overexpressing IL-10, which suggests a synergistic effect of IL-10 and Zn in the amelioration of the disease (Figure 2B). The DAI encompasses three scores, one of which is the weight loss. Relatively to control animals, both pMT-10 and BL/6 mice fed with Zn prior to DSS administration showed less weight loss (Figure S2 in Supplementary Material). pMT-10 mice preexposed to IL-10 showed the least reduction in colon length as compared to all control groups (pMT-10 or BL/6 fed with control water, and BL/6 fed with Zn-enriched water – Figure 2C), in line with maximal protection being conferred by IL-10. Histologic analysis of the organ, comprising the analysis of inflammatory infiltrates, architectural distortion (crypt shortening and branching) and ulceration, showed an improvement in pMT-10 mice preexposed to IL-10 as compared to BL/6 preexposed to Zn (Figures 2D,E). However, despite a reduction in the histological score of some pMT-10 mice preexposed to IL-10, the overall group did not reach statistical significance when compared to the other control groups (Figures 2D,E).

Thus, our data showed that IL-10 overexpression prior to intestinal insult afforded a significant degree of protection from DSS-induced colitis. Additionally, our data also suggest a synergistic effect of IL-10 and Zn in the amelioration of the disease. To investigate this issue, we repeated the experiment using pMT-10 mice crossed with IL-10Rα^−/−^ mice. Since pMT-10.IL-10Rα^−/−^ double mutant mice are unresponsive to IL-10, the effects observed would only be due to Zn administration. In these mice, we observed an accelerated disease progression upon DSS administration, with an elevated DAI score as early as day 3, in line with the known role of IL-10 in controlling the disease (Figure 2F). In these mice, Zn administration did not confer protection against DSS-induced colitis (Figure 2F). Taken together, our findings support the notion that the protection conferred by Zn requires IL-10 signaling.

### Preexposure to IL-10 Promotes a More Controlled Inflammatory Response

**Figure 3:**
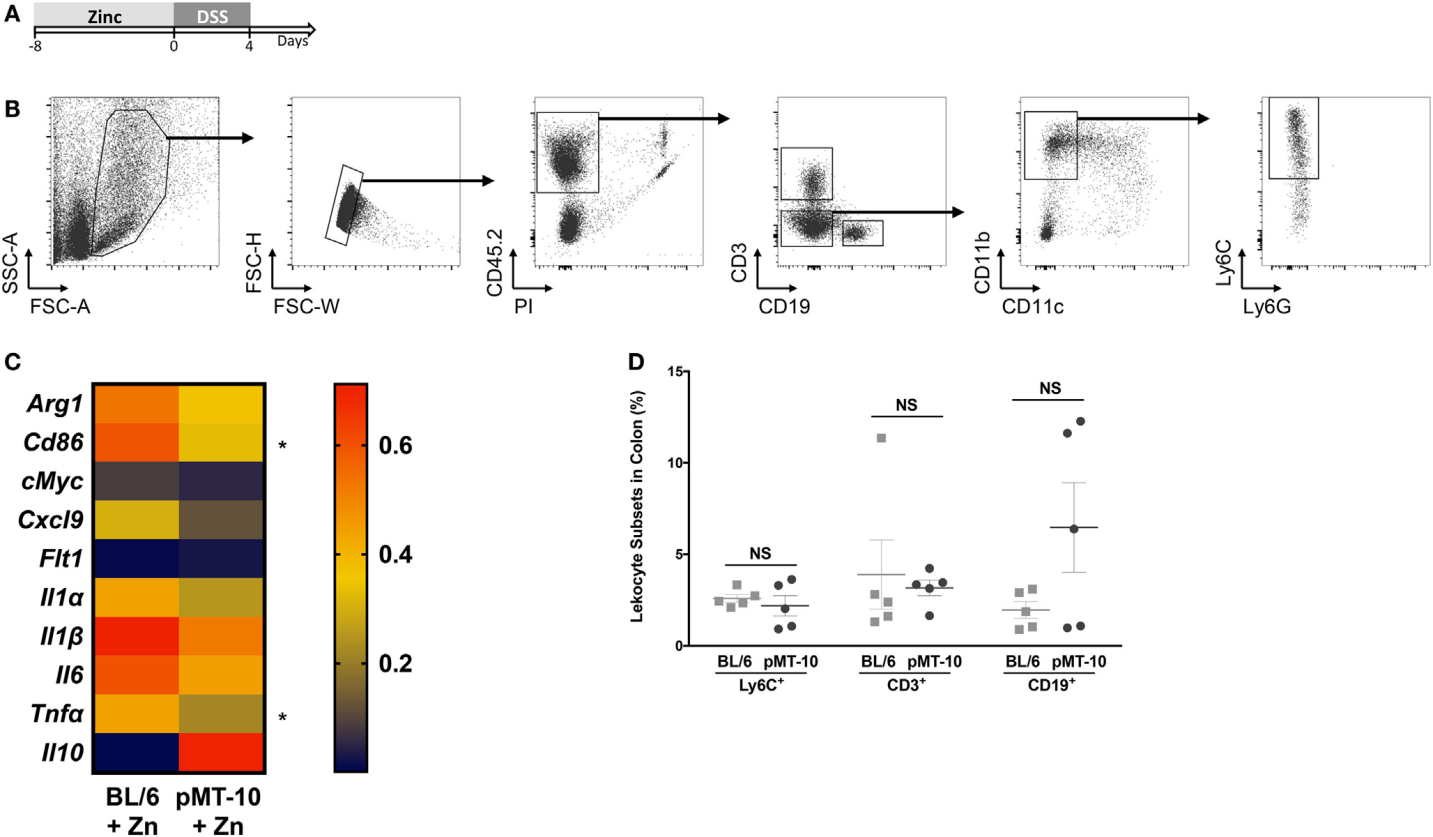
Ly6C^+^ cells preexposed to interleukin (IL)-10 reveal a less inflammatory profile upon DSS-induced colitis than those preexposed to Zn. **(A)** pMT-10 or BL/6 mice were fed with Zn-enriched water for 8 days, followed by 4 days of 3% DSS administration. **(B)** At the end of the DSS treatment, Lamina propria leukocytes (LPLs) were isolated and Ly6C^+^ cells sort-purified. Shown is the gating strategy for Ly6C^+^ cells purification. **(C)** Sort-purified Ly6C^+^ cells (*n* = 25 cells) were analyzed by qRT-PCR for a total of 22 genes using the BioMark HD system. Samples were normalized for *Hprt* expression. Represented is the expression heatmap compiling the genes which expression was detected in either mouse group. Each heatmap rectangle represents the mean of gene expression obtained for cells isolated from five independent mice. **(D)** The frequency of the different leukocyte subsets was determined upon staining of LPLs for Ly6C^+^ cell sorting. Each dot represents one independent animal; represented is also mean ± SEM. Data were analyzed with Student’s *t*-test, *p < 0.05.

Previous studies have shown that monocytes and macrophages are the major effector subsets of colonic inflammation (34, 47). Mice with macrophage-specific IL-10R deficiency develop a spontaneous colitic profile, emphasizing the importance of IL-10 in regulating the macrophage response to prevent uncontrolled inflammation (34). Thus, we next investigated whether IL-10 ameliorated DSS-induced colitis by restricting the monocyte/macrophage response. Considering that Zn administration also improved the outcome of DSS-induced colitis in BL/6 mice, we compared the transcriptional profile of monocytes/macrophages from BL/6 or pMT-10 mice preexposed to Zn and subjected to DSS administration for 4 days. We chose this time point, since signs of colitis induced by DSS in both BL/6 and pMT-10 mice only become obvious after day 4 of DSS administration. Thus, BL/6 and pMT-10 mice were fed with Zn-enriched water for 8 days and then received DSS for 4 days (Figure 3A). At this time point, Ly6C^+^ cells from each mouse from the different groups were FACS purified (Figure 3B). Expression of 22 genes (Table S1 in Supplementary Material) associated with the uncontrolled immune response developed in IBD were analyzed by multiplex RT-PCR. All samples, from both groups, expressed three house-keeping genes (*Hprt, Actb*, and *Gapdh*). Of the 22 genes analyzed, we failed to detect expression of 9 (*Il4, Il9, Il12α, Il12β, Il13, Il17, Il23, Ifnγ*, and *Cx3cl1)* in Ly6C^+^ cells isolated from the *lamina propria* in both groups. We detected expression of the 10 remaining genes in Ly6C^+^ cells, in Zn-fed BL/6 and pMT-10 mice after DSS administration (Figure 3C), but no expression in the absence of insult (data not shown). Thus, Ly6C^+^ cells alter their expression profile in response to DSS insult. Most interestingly, on day 4 post-DSS administration, Ly6C^+^ cells isolated from induced pMT-10 mice presented an overall less inflammatory profile than those isolated from BL/6 mice (Figure 3C). In the case of *Tnfα* and *Cd86*, the differences observed between the two mouse groups were statistically significant (Figure 3C). In all, these findings suggest that exposure to IL-10 before DSS induction acts by preventing an inflammatory profile in Ly6C^+^ cells. Of note, the frequency of inflammatory macrophages recruited to the inflamed gut was similar between the two groups, and the same was true for CD3 T cells and CD19 B cells, showing that IL-10 overexpression does not impact the recruitment of immune cells to the gut (Figure 3D).

### IL-10 Protection against DSS-Induced Colitis Is Not Long Lasting

**Figure 4:**
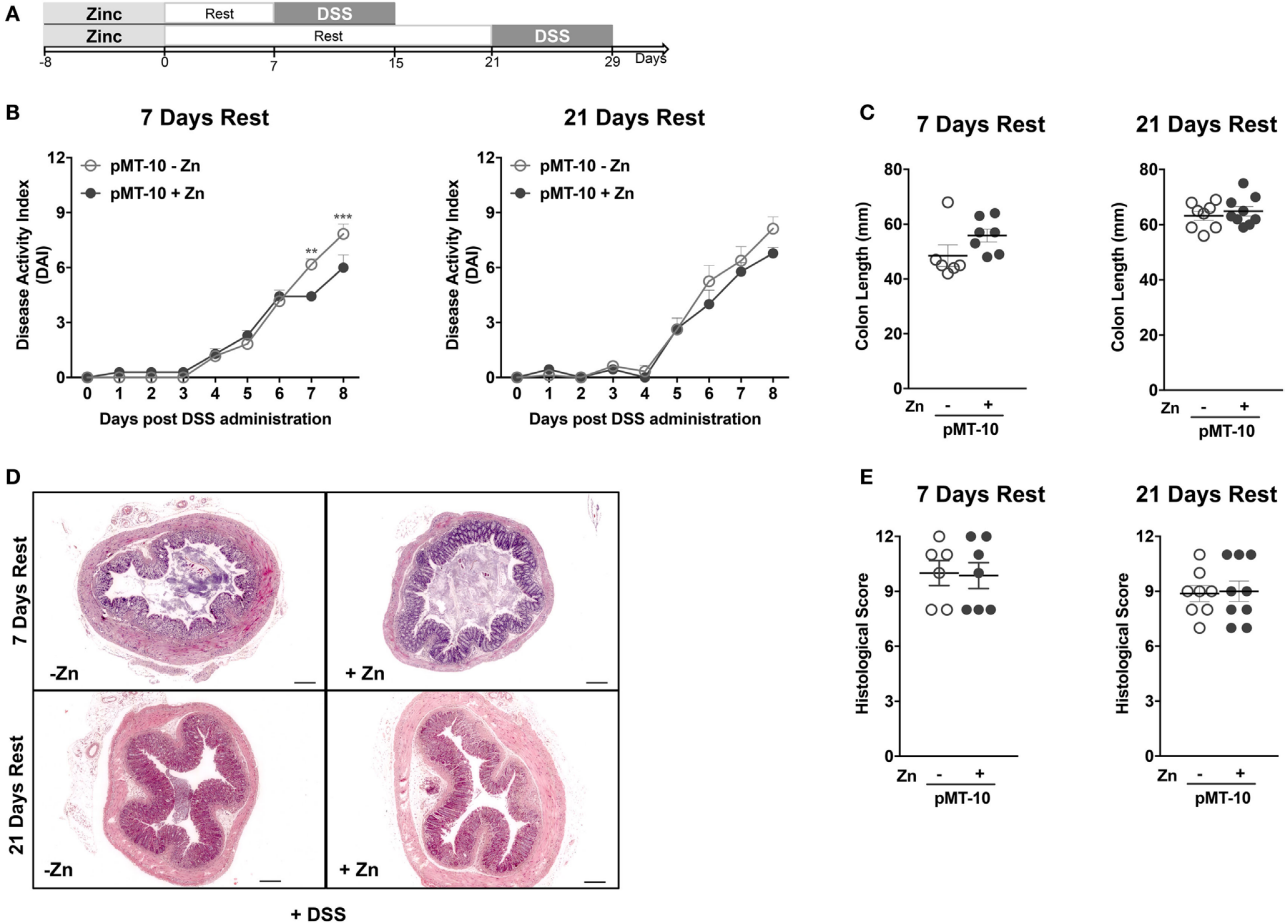
The interleukin-10 protection conferred against DSS-induced colitis is not long lasting. **(A)** pMT-10 mice were fed with control (pMT-10-Zn) or Zn-enriched (pMT-10 + Zn) water for 8 days, followed by a 7- or 21-day resting period where only normal water was available, and by 8 days of 3% DSS. **(B)** Disease progression based on Disease Activity Index (DAI) parameters was registered every day for 8 days. **(C)** Colon length measurement at day 8 of DSS administration. **(D)** Representative H&E-stained sections of large bowel at 40× magnification (scale bar = 200 µm). **(E)** Colitis scores derived from evaluation of colon and cecum from both groups. Each dot represents one independent animal, in two independent experiments; represented is also mean ± SEM. Data were analyzed with **(B)** two-way analysis of variance (Sidak’s multiple comparisons test) or **(C,E)** Student’s *t*-test.

In our mouse model, IL-10 is also overexpressed in the BM and is found at high levels in the serum, possibly creating an anti-inflammatory environment that could precondition *de novo* generated or circulating monocytes. In this setting, the circulating monocytes could thus be educated to be less responsive once recruited to the colon during DSS-induced colitis. To study this possibility, we combined a period of IL-10 overexpression with a resting period of 7 or 21 days prior to DSS administration (Figure 4A). As shown in Figure 1B, the levels of IL-10 return to basal ones as soon as 24 h after the zinc administration is suspended. Thus, after a 7-day rest, the circulating levels of IL-10 are normalized. Furthermore, we chose the 21 days time-point, because previous reports showing that resident intestinal macrophages have a life span of approximately 3 weeks (48). After 7-day rest period, the protection in DAI afforded by previous IL-10 exposure was partly lost (Figure 4B), being less pronounced than when no resting was performed and only observed at later time-points. After the 21-day resting period, the protection in DAI of pMT-10 previously overexpressing IL-10 was completely ablated (Figure 4B). Furthermore, independently of the resting period, no differences were observed between the experimental groups in what concerned colon length (Figure 4C) or histology (Figures 4D,E). Thus, we conclude that IL-10 overexpression, over a period of 8 days, does not confer long-lasting protection against intestinal inflammation.

## Discussion

Despite the fact that IBD is a treatable condition, there are many limitations to the therapeutic approaches currently available (5). Major obstacles in this context are the heterogeneity of the disease, which implies that dosage and schedule may differ across disease conditions, and the requirement of a localized action of the therapeutic agent. In view of the strong immune component associated with disease development, it is not surprising that therapeutic manipulations of the immune response have been widely sought approaches to tackle IBD. Indeed, a commonly used therapy for IBD is the administration of anti-tumor necrosis factor alpha (TNF-α) antibodies (49). However, in line with the above mentioned limitations, up to one-third of IBD patients do not respond to this therapy, and those who respond eventually develop some degree of intolerance to the medication (50). In this context, several animal models of IBD, both spontaneous and experimentally induced (such as DSS), were developed to investigate the role of various factors on the pathogenesis of the disease and to evaluate the different therapeutic options. A molecule that has been widely studied in the context of IBD is IL-10. This cytokine keeps intestinal inflammation in check by exerting a direct effect on monocyte/macrophage populations (34). Thus, it is not surprising that IL-10-based therapies have been tested in IBD. However, both in human (51–54) and mouse models (38), administration of IL-10 did not significantly improve intestinal inflammation, perhaps in part due to the fact that administered IL-10 did not reach the inflamed tissue.

In this study, we report a novel transgenic mouse model of inducible IL-10 overexpression, the pMT-10 mice, in which high IL-10 transcription is observed in the intestine, skin and BM. Upon induction of the transgene, high levels of IL-10 are detected also in the serum. Taking advantage of this novel mouse model, we investigated the dynamics of IL-10 afforded protection during DSS-induced colitis. We found that induction of IL-10 prior to DSS administration impacted the progression of colitis. We show that a short period of IL-10 overexpression before the induction of colitis ameliorated the disease outcome, despite the presence of CD11b^+^ Ly6C^+^ cells in the gut, previously associated with the development of detrimental inflammation. However, in comparison to control animals that do not overexpress IL-10, Ly6C cells isolated from the lamina propria of colitic pMT-10 mice showed a decreased inflammatory profile. Thus, we hypothesize that IL-10 overexpression impairs the response of these cells to the insult, reaffirming both the critical role of these cells on intestinal inflammation (47) and that of IL-10 in regulating their inflammatory responses (34). In line with a previously described protective role for Zn in the context of intestinal inflammation (55, 56), we show some effect of Zn in reducing the severity of colitis, which occurred both later and to a lower extent than that observed for the combined condition Zn + IL-10. In addition, the protective effect of Zn failed to overcome the exacerbated colitis observed in mice that did not respond to endogenous IL-10. The mechanistic bases underlying the protection conferred by Zn alone remain unknown. One possibility is that Zn may contribute to diminish the amount of free radical species generated during acute colitis which contribute to protein, DNA chain and lipid damage (57). In any case, as IBD patients often present a Zn deficiency and respond well to Zn supplementation therapy (58), the exploitation of combined IL-10 and Zn therapies may be worth considering. In line with this, the benefits of combined Zn and anti-TNF therapy were previously described (55).

The pMT-10 mouse model allows for local, as well as systemic, IL-10 overexpression. The fact that we detected increased transcription of the IL-10 transgene in the BM and elevated levels of seric IL-10 led us to hypothesize that preexposure to IL-10 might induce long-lasting transcriptional changes in circulating monocytes, for example through epigenetic imprinting. If this were the case, we might be able to educate these cells to gain long-lasting tolerance to DSS-induced colitis. Our data obtained after a 1- or 3-week rest post-IL-10 exposure show that recent IL-10 exposure is required for maximal protection. Thus, the protective effects of IL-10 were not sustained over time, implying that IL-10 presence at the time of insult is necessary to prevent colitis. Therefore, inducing IL-10 expression in our mouse model at the beginning of disease would be of interest. Unfortunately, we were unable to explore this possibility because the Zn necessary to activate the transgene precipitates in the presence of DSS, when both are provided in the drinking water. In addition, we were unable to induce high levels of IL-10 expression in the serum of mice fed with a Zn-enriched diet and ethical issues prevented us from attempting to induce sustained high levels of IL-10 by frequent gavage or i.p. injections of Zn-containing preparations.

In conclusion, we herein present a novel mouse model of inducible IL-10 overexpression. We also show the potential of this model for the study of the IL-10 biology in the specific setting of DSS-induced colitis. Our data further support the protective role for IL-10 in intestinal inflammation, showing that this cytokine delays disease progression even when delivered before DSS administration. However, the effect is not long-lasting, which calls for alternative approaches to prevent IBD.

## Ethics Statement

In Portugal, all animal experiments were performed in strict accordance with recommendations of the European Union Directive 2010/63/EU and previously approved by Portuguese National Authority for Animal Health–Direção Geral de Alimentação e Veterinária (DGAV). Mice were euthanized by CO2 inhalation with efforts to minimize suffering. In France, all animal procedures were approved by the Pasteur Institute Safety Committee and conducted according to French and European Community Institutional guidelines. Mice were euthanized by CO_2_ inhalation with efforts to minimize suffering.

## Author Contributions

AC, AM, and GC performed the experiments. AGC and PV made the pMT-10 mice. VM sequenced the genome of the pMT-10 mouse. AC, AM, AGC, IC, AC, PV, and MS planned the experiments and analyzed data. AC, AGC, PV, and MS wrote the article.

## Conflict of Interest Statement

The authors declare that the research was conducted in the absence of any commercial or financial relationships that could be construed as a potential conflict of interest.
