# Supplementary material for: The Dynamics of Interleukin-10-Afforded Protection during Dextran Sulfate Sodium-Induced Colitis
Source: Front Immunol. 2018 Mar 1;9:400. doi: 10.3389/fimmu.2018.00400 (PMC5837963; doi:10.3389/fimmu.2018.00400)
Supplement: Supplementary file 1 [file data_sheet_2.docx]

Supplementary Material

**The Dynamics of Interleukin-10-Afforded Protection during Dextran Sulfate Sodium-induced Colitis**

Ana Cardoso^1,2,3,4,5,6,7^, A. Gil Castro^6,7¶^, Ana Catarina Martins^1,2¶^, Guilhermina M. Carriche^1,2#a^, Valentine Murigneux^8,#b^, Isabel Castro^6,7^, Ana Cumano^3,4,5^, Paulo Vieira^3,4,5&^ and Margarida Saraiva^1,2&*^

*** Correspondence:** Margarida Saraiva: Margarida.Saraiva@ibmc.up.pt


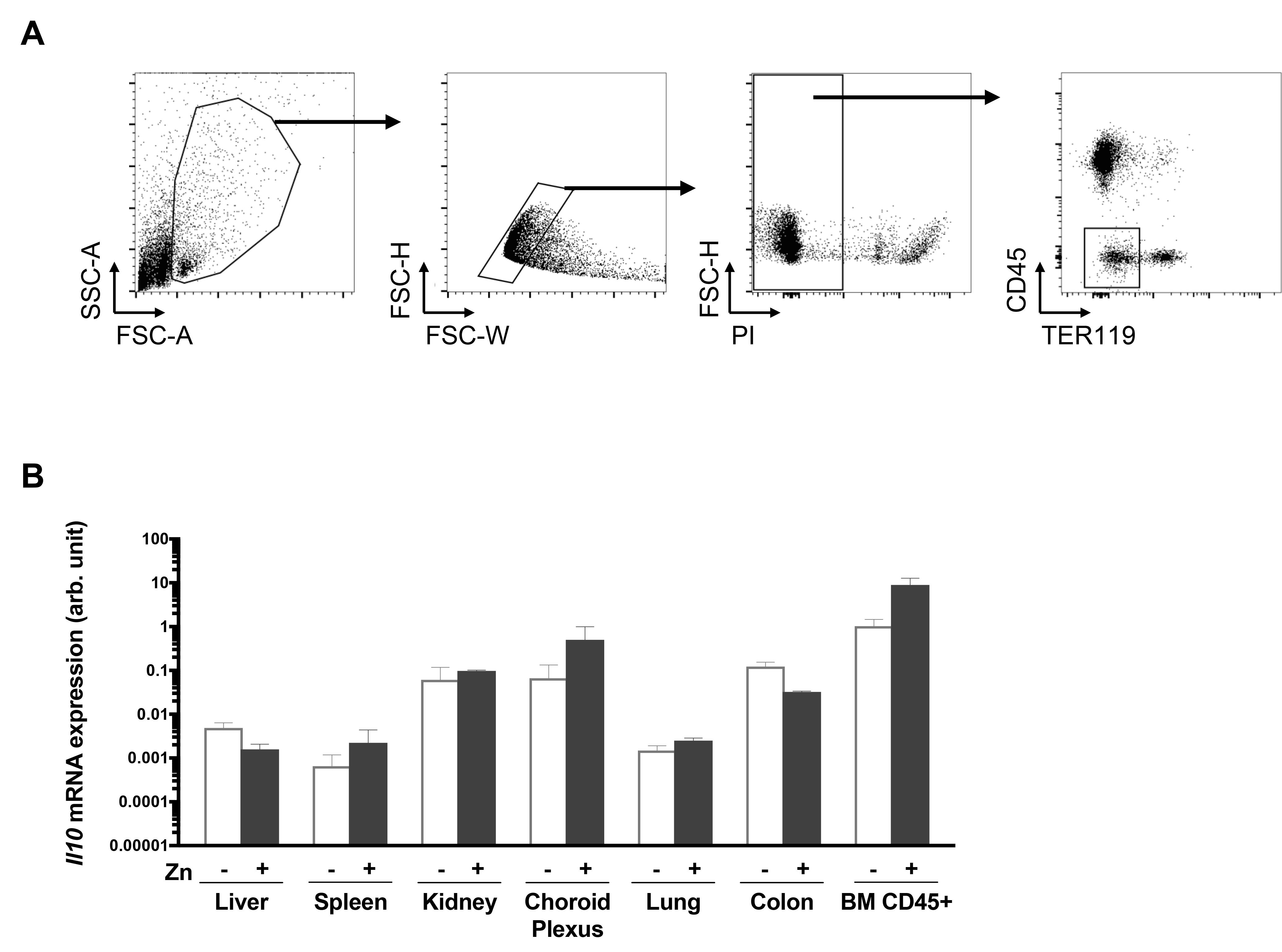


***Gate 1***

***Gate 2***

**Supplementary Figure 1. (A) Gating strategy for CD45^-^ TER119^-^ cells sort purification.** Live non-hematopoietic (CD45-TER119-, gate 1) and hematopoietic (CD45+, gate 2) cells were sort purified based on the lack of expression of CD45 and Ter119. **(B)** Cell suspensions were prepared from the indicated organs as specified in the Methods section, from pMT-10 mice fed with normal (-) or Zn-enriched (+) water. *Il10* mRNA expression was measured by qPCR as indicated in the Methods section.


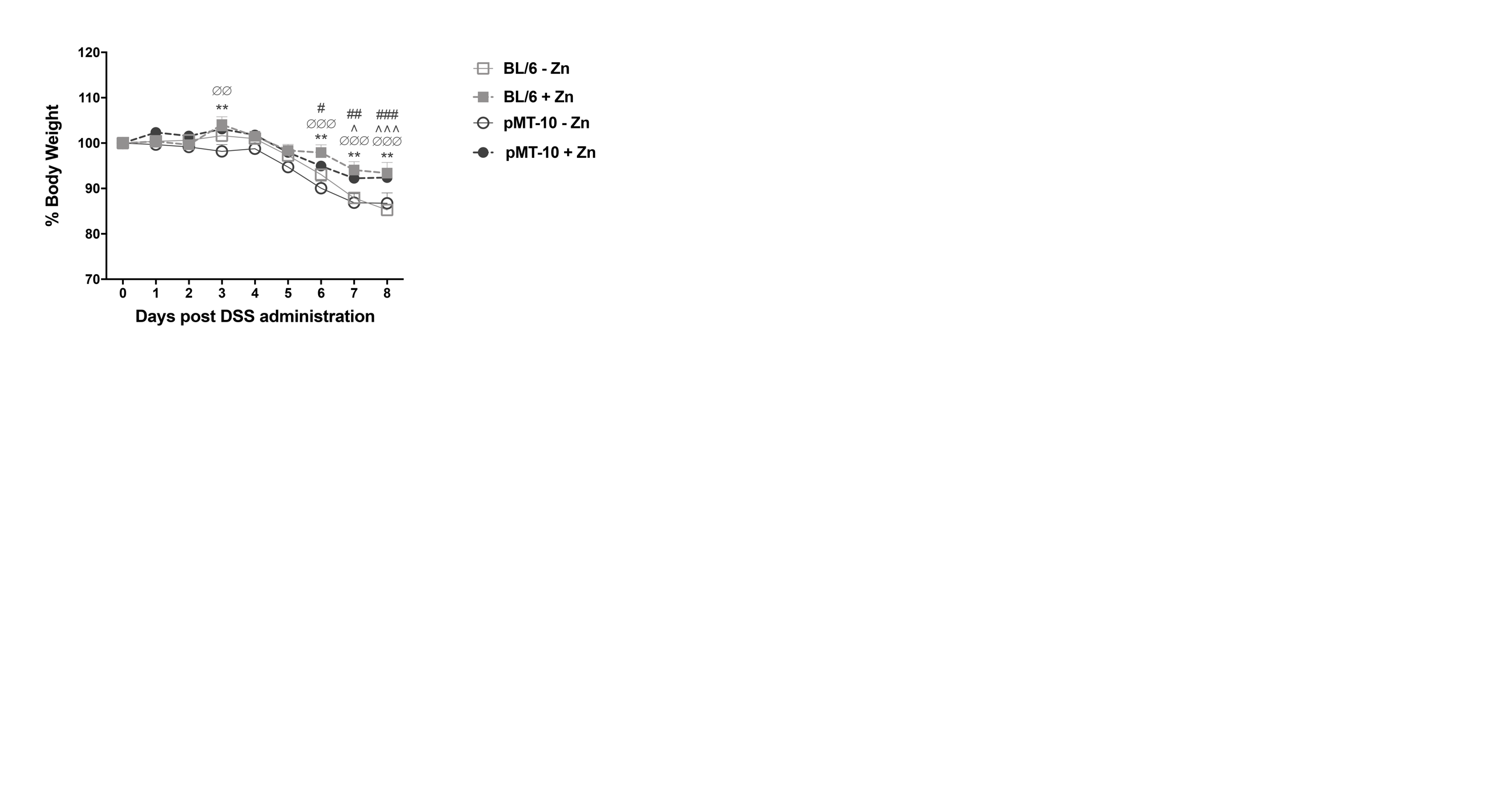


**Supplementary Figure 2.** **Progression of weight loss in DSS-induced BL/6 and pMT-10 mice.** BL/6 and pMT-10 mice, fed with control (-Zn) or Zn-enriched water (+Zn), were administered, for 8 days, 3% DSS in the drinking water. Body weight was registered every day for 8 days. Data were analysed with two-way ANOVA (Sidak's multiple comparisons test). # compare BL/6-Zn against BL/6+Zn; ^ compare BL/6-Zn against pMT-10-Zn; ∅ compare BL/6+Zn against pMT-10-Zn; * compare pMT-10-Zn against pMT-10+Zn. 1 symbol, p<0.05; 2 symbols p<0.01; 3 symbols, p<0.001.

**Supplementary Table 1.** List of Taqman assays used for the BioMark, related to Figure 4.

| Probe | Taqman Reference |
| --- | --- |
| *Hprt* | Mm03024075_m1 |
| *Gapdh* | Mm99999915_g1 |
| *Actb* | Mm02619580_g1 |
| *Il1α* | Mm00439620_m1 |
| *Il1β* | Mm00434228_m1 |
| *Il4* | Mm00445259_m1 |
| *Il6* | Mm00446190_m1 |
| *Il9* | Mm00434305_m1 |
| *Il10* | Mm01288386_m1 |
| *Il12α* | Mm00434169_m1 |
| *Il12β* | Mm01288989_m1 |
| *Il13* | Mm00434204_m1 |
| *Il17* | Mm00439618_m1 |
| *Il23* | Mm0110011_g1 |
| *Tnfα* | Mm00443258_m1 |
| *Ifnγ* | Mm01168134_m1 |
| *Cxcl1* | Mm00436454_m1 |
| *Arg1* | Mm00475988_m1 |
| *Cxcl9* | Mm00434946_m1 |
| *Cd86* | Mm00444543_m1 |
| *cMyc* | Mm00487804_m1 |
| *Flt1* | Mm01210866_m1 |
